# Stress-Induced Cardiomyopathy Mimicking Acute Coronary Syndrome in a High-Risk Female Patient: A Case Report

**DOI:** 10.7759/cureus.82618

**Published:** 2025-04-20

**Authors:** Zaid Al Hassani, Zahraa Al Haboobi, Jaafar Hasan, Yazan Katroon, Rahaf Wardeh

**Affiliations:** 1 College of Medicine, University of Sharjah, Sharjah, ARE; 2 Internal Medicine, Dubai Health, Dubai, ARE

**Keywords:** acute coronary syndrome, apical ballooning, postmenopausal women, stress cardiomyopathy, takotsubo cardiomyopathy

## Abstract

Takotsubo cardiomyopathy (TTC), also known as takotsubo syndrome, is a transient but potentially serious cardiac dysfunction that often mimics acute coronary syndrome (ACS) in the absence of obstructive coronary artery disease. It is typically associated with intense emotional or physical stress and presents predominantly in postmenopausal women, but can occur in other populations. We present a case of a 55-year-old postmenopausal woman with multiple cardiac risk factors, including uncontrolled diabetes, dyslipidemia, and smoking, who developed chest pain and dynamic troponin elevation (42 ng/L to 97 ng/L) following a severe emotional stressor. She was initially diagnosed with non-ST elevation myocardial infarction (NSTEMI) based on ischemic electrocardiographic changes and a rising troponin trend. Subsequent echocardiography revealed apical akinesis with basal hyperkinesis - features typical of TTC. The patient was initially managed as a case of NSTEMI, with treatment, including dual antiplatelet therapy (DAPT), statins, beta-blockers, angiotensin-converting enzyme (ACE) inhibitors, and insulin, with complete recovery of left ventricular ejection fraction (LVEF) during hospitalization. Mild diastolic dysfunction persisted at a five-month follow-up without clinical heart failure or the need for additional intervention. This case underscores the importance of maintaining clinical suspicion for non-ischemic causes such as stress-induced cardiomyopathy in patients presenting with ACS-like symptoms. This vigilance is crucial as standard ischemic evaluation is critical, and TTC is a diagnosis of exclusion. It requires careful assessment via imaging modalities, echocardiography, CT angiogram, and cardiac MRI to differentiate it from ACS or other cardiomyopathies, as management strategies differ significantly.

## Introduction

Takotsubo cardiomyopathy (TTC), also known as stress-induced cardiomyopathy or “broken heart syndrome,” is a transient cardiac condition that closely mimics acute coronary syndrome (ACS) [1,2]. First identified in Japan in 1990, the name “Takotsubo” derives from a Japanese octopus trap, which resembles the characteristic apical ballooning of the left ventricle in this condition - a hallmark deformity where the apex becomes akinetic and bulges during systole, while the base contracts typically [1].

TTC is seen predominantly in postmenopausal women, accounting for 80-90% of cases, but has been reported in men and younger individuals, albeit rarely [3,4]. It most often arises in response to acute emotional stressors (e.g., grief, interpersonal conflict, and even positive events, termed “happy heart syndrome”) [5] or physical stressors (e.g., acute illness, surgery, sepsis, and neurological injury) [6]. One study found that physical triggers were more common than emotional ones (36.0% vs. 27.7%), with 7.8% of patients experiencing both and 28.5% developing TTC without any identifiable trigger [7]. Several clinical conditions have been implicated in the development of a takotsubo-like syndrome, including infections like sepsis and a variety of neurological disorders such as subarachnoid hemorrhage, seizures, cerebrovascular accidents, brain neoplasms, and traumatic brain injuries [8]. Endocrine tumors like pheochromocytoma have also been recognized as potential triggers [9]. In addition, the administration of catecholamines, such as dopamine, dobutamine, epinephrine, and norepinephrine, particularly during procedures like cardiovascular stress testing, anesthesia, or other situations involving cardiovascular strain, may provoke similar cardiac dysfunction [10]. TTC represents 1-3% of suspected ACS presentations, making it a critical diagnostic consideration in patients with ACS-like symptoms, particularly those with identifiable stressors or atypical risk profiles [1].

Clinically, TTC presents with chest pain or dyspnea indistinguishable from ACS, often accompanied by electrocardiographic (ECG) abnormalities such as ST-segment deviations, diffuse T-wave inversions (frequently evolving over days), and a modestly prolonged QTc interval (≥480 ms) [7,11]. Cardiac biomarkers, such as troponin and creatine kinase-myocardial band (CK-MB), are typically elevated but to a lesser degree than in ST elevation myocardial infarction (STEMI) [7]. Due to this overlap, coronary angiography remains the gold standard to exclude obstructive coronary artery disease, while left ventriculography or advanced imaging (e.g., cardiac magnetic resonance) is critical for confirming transient ventricular dysfunction and ruling out irreversible myocardial injury, according to the International Expert Consensus Document on Takotsubo Syndrome (Part II) [12]. However, echocardiography plays a vital early role by revealing the hallmark wall-motion abnormalities (e.g., apical akinesis with basal hyperkinesis), which can raise suspicion for TTC even before angiography [12]. Notably, the consensus highlights the utility of the InterTAK Diagnostic Score, a tool integrating clinical parameters such as female sex, emotional/physical triggers, and absence of ST-segment depression to stratify patients for invasive evaluation [12].

## Case presentation

A 55-year-old postmenopausal woman with a history of poorly controlled type 2 diabetes mellitus (hemoglobin A1c: 12.5%), dyslipidemia, hypothyroidism (on levothyroxine), non-alcoholic fatty liver disease (NAFLD), and active smoking (three to four cigarettes per day) presented to the emergency department with sudden, severe left-sided chest tightness. The pain began shortly after an emotionally intense family conflict earlier that day, leaving her in a vulnerable state. She denied significant dyspnea, lightheadedness, or nausea. The chest pain was persistent, and she had not experienced similar symptoms previously. She had no known history of hypertension, coronary artery disease, or cardiomyopathy, and no family history of cardiomyopathy or premature coronary disease.

**Figure 1:**
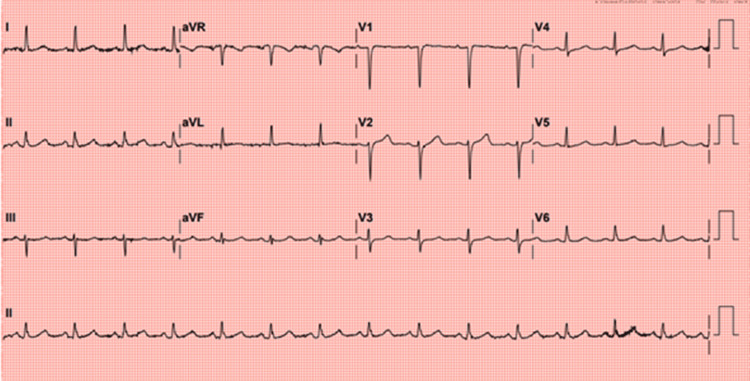
Initial electrocardiogram at presentation showing normal sinus rhythm without acute ischemic changes. Electrocardiogram (ECG) obtained at presentation demonstrated normal sinus rhythm without ST-segment elevations, ST depressions, or T-wave inversions.

On presentation, her vital signs were within normal limits (Table 1), and her oxygen saturation was 98% on room air. There were no signs of heart failure or arrhythmia on the exam. Her random blood glucose was 500 mg/dL on admission, indicating hyperglycemic urgency. This was managed with subcutaneous insulin and intravenous fluids. Initial electrocardiogram (ECG) showed normal sinus rhythm without acute ischemic changes (Figure 1) - no ST-segment elevations, ST depressions, or T-wave inversions were present. Given her ongoing chest pain and multiple risk factors, she was treated as an ACS patient: dual antiplatelet therapy (DAPT), a high-intensity statin (atorvastatin 80 mg), subcutaneous enoxaparin, and beta-blocker therapy were initiated. Serial laboratory tests showed rising cardiac biomarkers consistent with myocardial injury (Table 2): troponin-T increased from 42 ng/L at admission to 97 ng/L four hours later (normal <14 ng/L), fulfilling criteria for non-ST elevation myocardial infarction (NSTEMI), and creatine kinase-myocardial band was mildly elevated (Table 3). Repeat ECGs over the next 24 hours demonstrated evolutionary changes.

**Table 1:** Initial vital signs recorded in the emergency department reflecting hemodynamic stability.

Vital sign	Measurement
Blood pressure	119/83 mmHg
Heart rate	78 beats/min
Respiratory rate	18 breaths/min
Temperature	36.9°C
Weight	75 kg
Height	163 cm

**Table 2:** Serial troponin T levels.

Date/Time	Troponin T (ng/L)	Reference range & units
April 4, 2023, 08:09	42 (H)	<14 ng/L
April 4, 2023, 10:37	97 (H)	<14 ng/L
April 5, 2023, 05:13	37 (H)	<14 ng/L

**Table 3:** Cardiac markers (CK-MB levels). CK-MB: creatine kinase-myocardial band.

Component	CK-MB (mass)	Reference range & units
April 4, 2023, 10:37	4.8	<4.89 ng/mL
April 4, 2023, 11:35	5.2 (H)	<4.89 ng/mL
April 5, 2023, 05:13	37 (H)	<4.89 ng/mL

**Figure 2:**
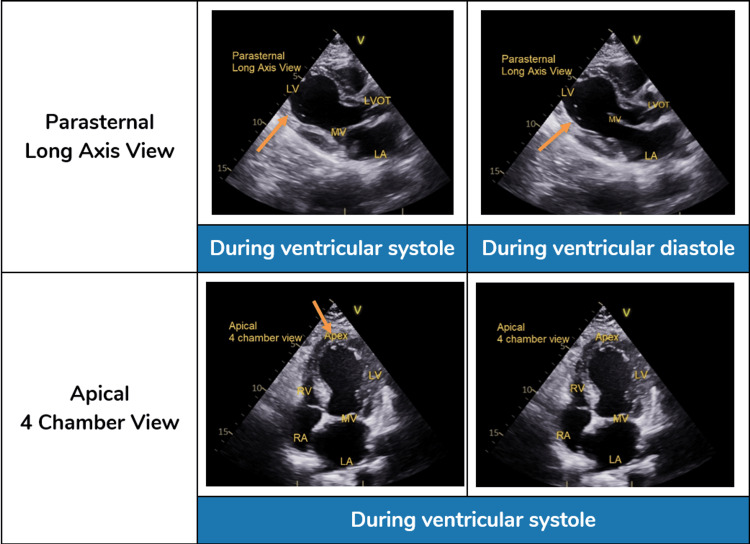
Admission transthoracic echocardiogram demonstrating classic features of takotsubo cardiomyopathy. Initial transthoracic echocardiogram (TTE) performed at admission demonstrated mid-to-distal left ventricular (LV) hypokinesis with apical ballooning, preserved basal contractility, and severely reduced global left ventricular (LV) systolic function (left ventricle ejection fraction: 25–30%), alongside normal left ventricular (LV) dimensions (end-diastolic diameter: 48 mm), mild diastolic dysfunction (E/e’ ratio: 12), and trace mitral regurgitation, hallmarks of takotsubo cardiomyopathy. LV: left ventricle; LA: left atrium; LVOT: left ventricular outflow tract; MV: mitral valve; RA: right atrium; RV: right ventricle.

**Figure 3:**
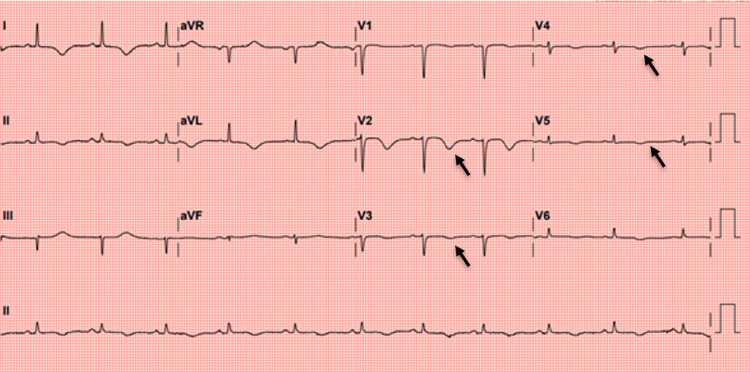
Day two follow-up electrocardiogram demonstrating QT interval prolongation and anterior T-wave inversions. Electrocardiogram (ECG) performed on day two showed a prolonged corrected QT (QTc) interval and deep T-wave inversions in the anterior precordial leads.

Transthoracic echocardiography (TTE) was performed within 10 hours of presentation. It revealed mid-to-distal left ventricle (LV) akinesis with apical ballooning and a severely reduced left ventricle ejection fraction (LVEF) of 25-30%, with preserved basal contraction (Figure 2). These findings were suggestive of TTC, although an acute ischemic event had not been ruled out at that early juncture. The TTE showed normal LV chamber dimensions and only trace mitral regurgitation, but did note mild diastolic dysfunction (impaired relaxation) and an incidental false tendon in the LV apex. No left ventricular thrombus was seen. The working diagnosis remained NSTEMI, but stress-induced cardiomyopathy was considered, given the wall-motion pattern disproportionate to the distribution of any single coronary artery. By hospital day two, her ECG revealed a prolonged corrected QT interval (QTc: 474 ms) with deep T-wave inversions in the anterior precordial leads (Figure 3), changes often seen in TTC but also possible in ischemia.

Laboratory investigations (Table 4) highlighted uncontrolled diabetes (glycosylated hemoglobin (HbA1c): 12.5%), dyslipidemia (low-density lipoprotein: 163 mg/dL), and elevated liver enzymes. Thyroid function tests were within normal limits. In light of this QT prolongation, electrolyte levels were closely monitored and maintained within normal limits (potassium: 3.8 mEq/L, sodium: 135 mmol/L, ionized calcium: 1.18 mmol/L) to mitigate arrhythmic risk; no ventricular arrhythmias occurred. There were no clinical or laboratory indicators of myocarditis (CRP = 2.4 mg/L). Erythrocyte sedimentation rate was not assessed, and pro-B-type natriuretic peptide (proBNP) was found to be <10 pg/mL.

**Table 4:** Abnormal laboratory results highlighting dyslipidemia and poor glycemic control. The lipid profile revealed several abnormalities, including elevated total cholesterol (246 mg/dL), triglycerides (214 mg/dL), low-density lipoprotein (LDL) cholesterol (163 mg/dL), and non-high-density lipoprotein (HDL) cholesterol (206 mg/dL), all of which exceed recommended thresholds, indicating significant dyslipidemia. Additionally, the glycosylated hemoglobin (HbA1c) value of 12.5% is markedly elevated, reflecting poor long-term glycemic control and indicating uncontrolled diabetes. SGPT: serum glutamic pyruvic transaminase; ALT: alanine aminotransferase; TSH: thyroid-stimulating hormone.

Component	Value	Reference range & units
Lipid profile		
Total cholesterol fasting	246 (H)	<190 mg/dL
Triglycerides	214 (H)	<150 mg/dL
HDL-cholesterol	40 (L)	48 mg/dL
LDL-cholesterol	163 (H)	<115 mg/dL
Non-HDL cholesterol	206 (H)	<145 mg/dL
Glucose		
HbA1c	12.5 (H)	<5.7%
Liver function test		
Bilirubin, total	0.2	0 - 1.0 mg/dL
Alkaline phosphatase	167 (H)	35 - 104 U/L
SGPT (ALT)	24	0 - 31 U/L
Total protein	7.2	6.6 - 8.7 g/dL
Albumin	4.1	3.4 - 4.8 g/dL
Globulin	3.1	2.8 - 3.4 g/dL
Thyroid function test		
Free T4	20.5	11.0 - 22.0 pmol/L
TSH	1.440	0.3 - 4.2 uIU/mL

**Figure 4:**
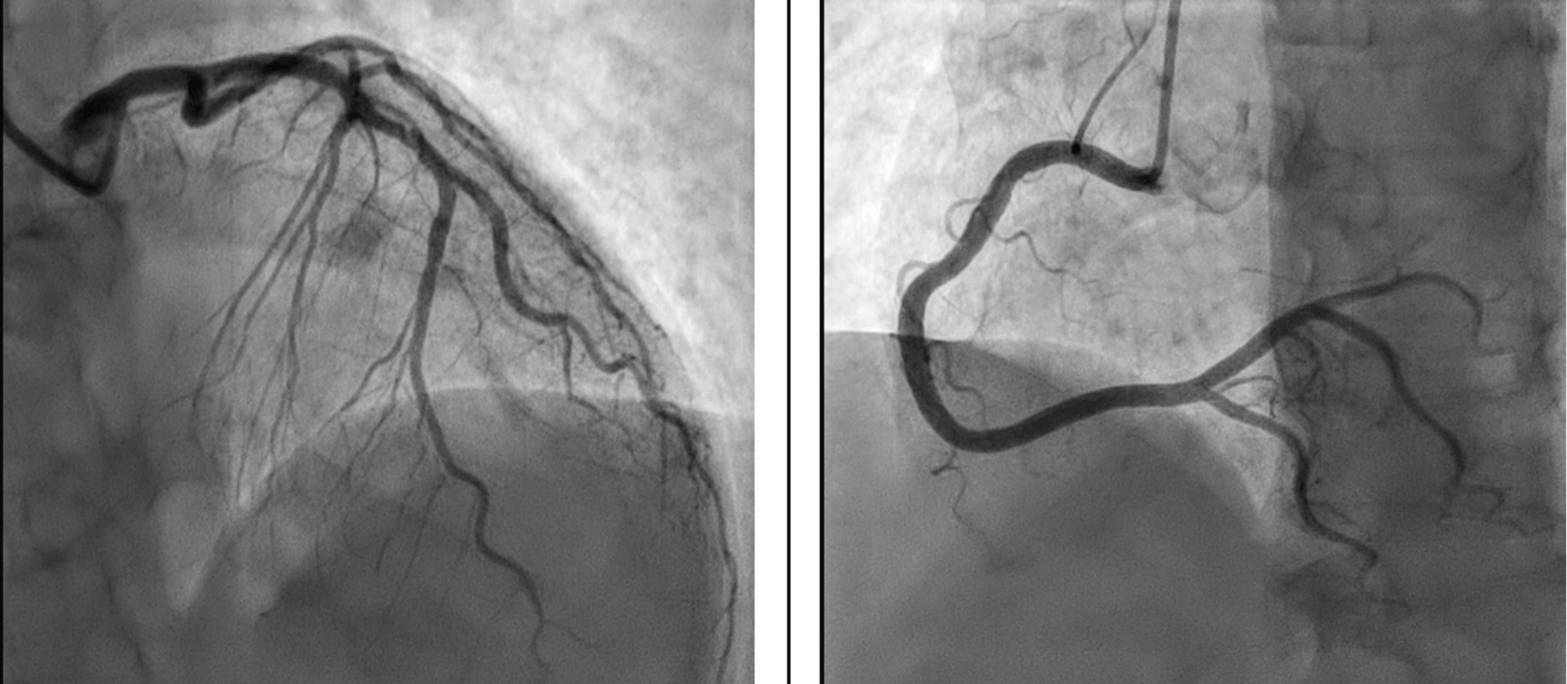
Coronary angiography demonstrating patent coronary arteries and absence of obstructive disease. Coronary angiography revealed widely patent coronary arteries without evidence of stenosis, thrombus, or plaque disruption in the left anterior descending (LAD) artery, left circumflex (LCX) artery, and right coronary artery (RCA). The angiogram confirmed right coronary dominance and effectively excluded obstructive coronary artery disease as the underlying cause of the observed left ventricular (LV) dysfunction.

The patient was managed conservatively in the coronary care unit. Beyond acute ACS protocol medications (antithrombotics and statins), she was started on guideline-directed heart failure therapy, including a beta-1 selective blocker (bisoprolol 2.5 mg daily) and an angiotensin-converting enzyme (ACE) inhibitor (lisinopril 2.5 mg daily) due to the severely reduced LVEF. Bisoprolol (2.5 mg daily) was chosen over carvedilol to minimize metabolic effects in her insulin-dependent diabetes. Her profound hyperglycemia was addressed acutely with insulin, and plans were made to intensify outpatient diabetes management after discharge. Her hemodynamics remained stable over the next 48 hours without vasopressor support, and her chest pain resolved. As ACS needed to be excluded, coronary angiography was requested; however, the procedure was initially postponed due to the patient's refusal and was performed only after she eventually consented. The delayed angiogram (Figure 4) demonstrated completely normal coronary arteries without any stenosis, definitively ruling out obstructive coronary artery disease. This timing was considered acceptable as the patient was clinically stable and low-risk for infarct-related complications under close monitoring.

**Figure 5:**
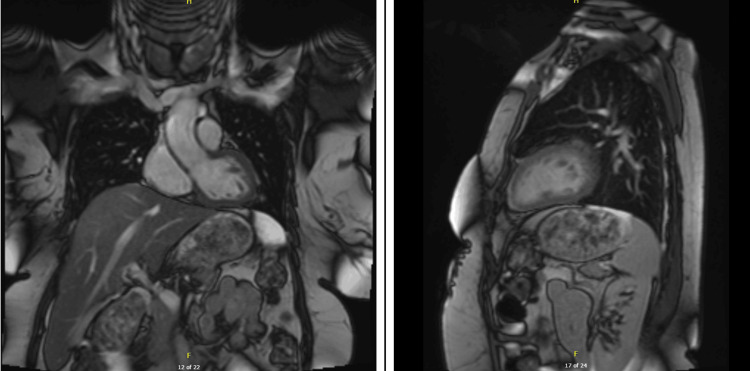
Follow-up cardiac magnetic resonance imaging (MRI) on day seven demonstrating functional recovery and absence of myocardial injury. Cardiac MRI performed on day seven demonstrated normalized left ventricular (LV) volumes (end-diastolic volume (EDV): 97 mL; indexed EDV: 54 mL/m²) and ejection fraction (EF) of 75%. There was no late gadolinium enhancement (LGE), ruling out myocardial infarction or fibrosis. A small area of isolated peri-myocardial enhancement was noted in the inferior basal wall, likely representing reactive changes. T1 and T2 parametric mapping values were within normal limits (T1 (septal): 1092 milliseconds; T2: 47 milliseconds), effectively ruling out myocarditis or infarction and supporting the diagnosis of stress-induced (takotsubo) cardiomyopathy.

Cardiac magnetic resonance imaging (MRI) was obtained on day seven to further evaluate myocardial function and tissue characteristics. The MRI (Figure 5) showed a normalized LVEF of 75%, indicating a dramatic recovery of systolic function. The previously akinetic apical segments contract normally (no residual apical ballooning). There were no findings of myocardial infarction or fibrosis on late gadolinium enhancement. A small focus of isolated peri-myocardial enhancement in the basal inferior wall was noted; this was thought to represent a benign reactive change or artifact, as T1/T2 mapping values were within normal limits.

The patient's hospital course was uncomplicated. By day seven, her cardiac biomarkers had normalized, and ECG changes were improving. She was discharged on bisoprolol, lisinopril, and her prior medications (including continuation of high-intensity statin and thyroxine). Oral hypoglycemic agents were adjusted, and insulin was prescribed for better glycemic control. She was advised to avoid intense emotional stress and referred for outpatient endocrinology and psychology follow-up for risk factor management and stress coping strategies.

**Figure 6:**
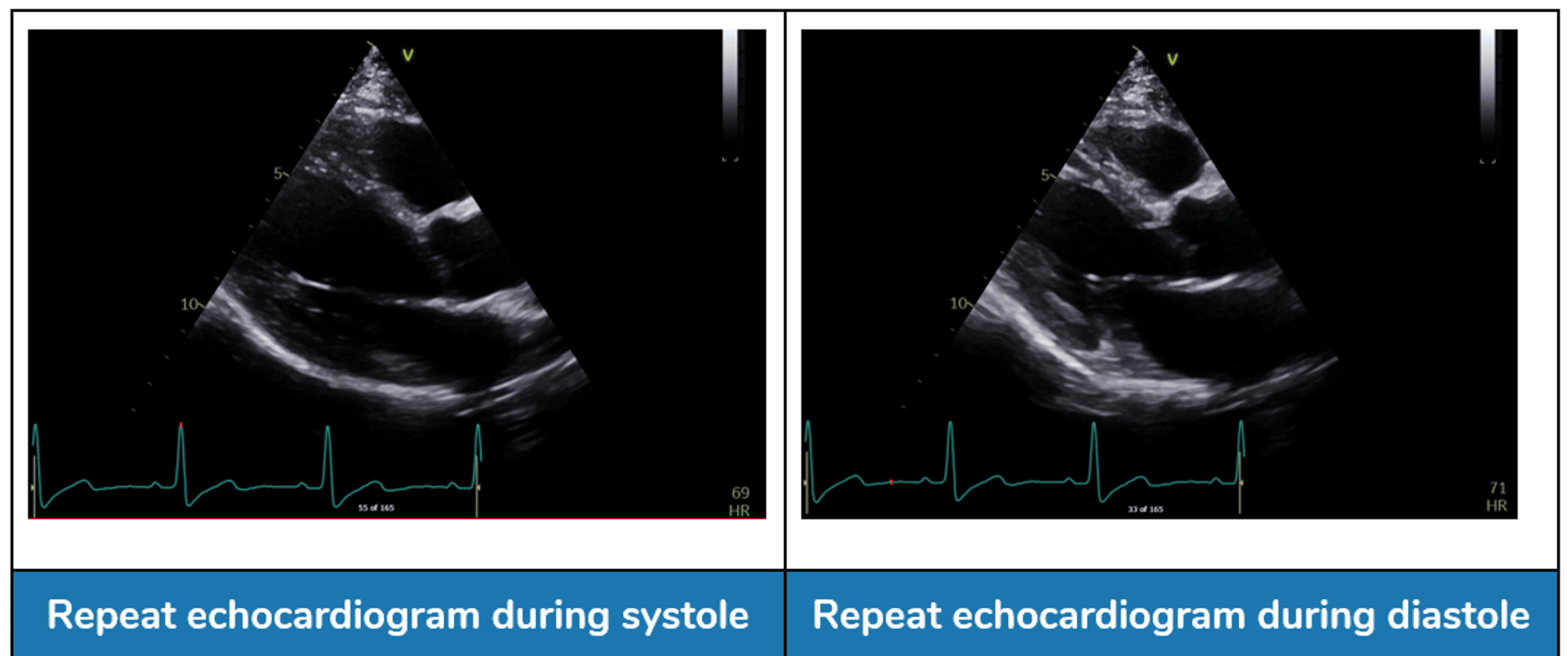
Repeat echocardiogram (five months after initial presentation). Repeat echocardiogram at five months post discharge showed significant recovery of left ventricular (LV) systolic function (biplane left ventricle ejection fraction (LVEF): 45–50%, triplane LVEF: 50%), with mild residual grade 1 diastolic dysfunction (E/A ratio: 0.8, deceleration time: 220 ms) and a persistent false tendon in the apical region, confirming the reversible nature of takotsubo cardiomyopathy.

On outpatient follow-up at five months, the patient was asymptomatic. Repeat echocardiography (Figure 6) showed LVEF of 45-50% and mild grade 1 residual diastolic dysfunction, which was asymptomatic and not associated with functional limitations. The incidental LV apical false tendon had no clinical correlation with symptoms or repolarization abnormalities. This slight decrease in LVEF compared to the MRI finding was not associated with any symptoms or signs of heart failure; it likely reflected differences between imaging modalities. The apical wall motion was normal, confirming that the cardiomyopathy had resolved. The patient's case demonstrates a classic TTC recovery and only minor diastolic relaxation abnormality, emphasizing the importance of follow-up imaging to assess for any lasting cardiac changes.

## Discussion

In this case report, we aim to highlight the diagnostic and therapeutic complexities of TTC, particularly when it presents features mimicking ACS. Although often underrecognized, TTC can lead to significant morbidity, including cardiogenic shock and cardiac arrest, particularly in postmenopausal women exposed to acute emotional or physical stressors [4].

Diagnosing TTC can be challenging due to its overlap with ACS, as illustrated in Table 5, which compares the distinguishing features of TTC and ACS. The revised Mayo Clinic criteria (2008) define TTC by four criteria: (1) transient regional wall-motion abnormality beyond a single vascular territory; (2) presentation mimicking myocardial infarction (e.g., chest pain, ECG changes, and modest troponin rise); (3) absence of obstructive coronary artery disease; and (4) exclusion of alternative diagnoses like myocarditis or pheochromocytoma [6]. Our patient fulfilled all of these criteria.

**Table 5:** Comparison of takotsubo cardiomyopathy (TTC) and acute coronary syndrome (ACS).

Feature	Takotsubo cardiomyopathy (TTC)	Acute coronary syndrome (ACS)
Demographics	Predominantly postmenopausal women	Older individuals with typical cardiac risk factors
Trigger	Emotional or physical stress (often acute), whether positive or negative emotions	Atherosclerotic plaque rupture, thrombus formation
Cardiac biomarkers	Mild to moderate troponin elevation	Typically elevated, proportional to infarct size
Echocardiogram	Regional wall motion abnormality, beyond a single vascular territory (non-coronary pattern)	Wall motion abnormality confined to the territory of the affected coronary artery
Coronary angiography	No obstructive coronary artery disease	Evidence of significant coronary artery obstruction
Left ventricular function	Transient dysfunction and apical ballooning; typically recovers in 1-4 weeks	Persistent dysfunction depending on infarct size

The underlying mechanism is thought to involve catecholamine-induced myocardial stunning, where excessive sympathetic stimulation causes transient LV dysfunction and apical akinesia, even in the absence of coronary artery disease [7]. This hypothesis is supported by our patient's rapid recovery in LV function, with her LVEF normalizing within one week, consistent with the typical one- to four-week recovery window. Although traditionally associated with negative emotional stressors, as in our patient's case, TTC can also follow positive events ("Happy Heart Syndrome"), underscoring the role of catecholamine surge regardless of emotional valence [5].

Pathophysiologic mechanisms include catecholamine-induced calcium overload, oxidative stress, and metabolic dysregulation in cardiomyocytes, resulting in regional wall-motion abnormalities [5,7]. Our patient's presentation and imaging matched the classic apical variant of TTC. Although the subtype does not alter initial management, prognostic differences exist. For example, reverse TTC may cause dynamic left ventricular outflow tract (LVOT) obstruction and shock, while apical variants carry a higher risk of thrombus formation due to regional akinesis [7]. LVOT obstruction and mural thrombus were ruled out by echocardiography in our patient.

TTC can lead to cardiogenic shock or cardiac arrest and is a recognized cause of transient cardiac dysfunction [12]. The most common variant, the "Takotsubo type," features basal hyperkinesis with apical ballooning. In addition to this classic form, three other atypical variants have been described: the mid-ventricular type (involving mid-LV ballooning), the localized type (with segmental akinesis), and the reverse variant (marked by basal akinesis and apical hypercontraction) [12,13]. All forms of TTC require close monitoring for potential complications, including arrhythmias, heart failure, and thromboembolism [12].

TTC management is mainly supportive, focusing on treating heart failure, preventing complications (e.g., LV thrombus and arrhythmia), and addressing precipitating factors. Long-term therapy remains debated. However, the current expert consensus supports using beta-blockers and ACE inhibitors or angiotensin receptor blockers (ARBs) for three to six months, even after normalization of LV function, to reduce sympathetic stimulation and aid myocardial remodeling [12,13]. Mineralocorticoid receptor antagonists may also be considered, particularly in patients with persistent LV dysfunction, due to their potential to counteract catecholamine-driven myocardial injury [12]. In uncomplicated cases, therapy is typically tapered after recovery. In contrast, patients with residual dysfunction or recurrent TTC episodes may require prolonged or indefinite therapy [13]. Our patient received a beta-blocker and an ACE inhibitor - standard therapy aimed at reducing adrenergic stress and facilitating ventricular recovery.

TTC carries a measurable risk of recurrence, reported between 1.5% and 10%, with an annual recurrence rate of approximately 1.8% per patient-year [12]. Similar or entirely different stressors may trigger a recurrence. Therefore, psychosocial support and stress management are integral to care. We referred our patient for psychiatric evaluation and counseling to help her develop healthier coping mechanisms for emotional stress.

Identifying psychological comorbidities such as anxiety or depression is increasingly viewed as essential to TTC management and may help reduce recurrence risk and enhance quality of life [13]. Our patient agreed to counseling. The lack of standardized management guidelines has led to variability in practice; some clinicians treat TTC similarly to ischemic cardiomyopathy, while others favor a more conservative, symptom-based approach [13].

Cardiogenic shock contributes significantly to early TTC mortality, affecting about 10% of cases [14]. While our patients had typical cardiac risk factors, many TTC patients did not, highlighting its unpredictability [14]. In a cohort of 214 TTC patients, life-threatening arrhythmias or cardiac arrest occurred in 10.7% [15]. For patients with cardiogenic shock, mechanical circulatory support (e.g., intra-aortic balloon pump and percutaneous ventricular assist devices) or calcium sensitizers (e.g., levosimendan) are preferred over adrenergic inotropes [15]. Fortunately, our patient did not require inotropes.

A key factor in TTC-associated cardiogenic shock is LVOT obstruction, which arises from the mitral valve's basal hyperkinesis and systolic anterior motion (SAM), often exacerbated by proximal septal hypertrophy in older adults [16]. Thromboprophylaxis is also important: in patients with severe LV dysfunction (e.g., LVEF <30%) or apical ballooning, the risk of mural thrombus formation is heightened [15]. Empiric anticoagulation is advised in such cases. We initiated low-dose heparin while our patient's LVEF was 25%, even before angiography, both as a thromboembolic precaution and as part of NSTEMI management. No thrombus developed, and anticoagulation was discontinued upon LV recovery.

The RETAKO Registry revealed that inotropic agents were more commonly used in non-survivors (59%) than survivors (11%), implicating both disease severity and catecholamine exposure in adverse outcomes [16]. Similarly, data from the International Takotsubo (InterTAK) Registry indicate that approximately 4.9% of TTC patients suffer cardiac arrest. Among these, ventricular fibrillation was the most frequent rhythm (44%), while ventricular tachycardia was the least (13.1%) [17]. The InterTAK Diagnostic Score aids early TTC suspicion prior to angiography using clinical features (e.g., female sex, emotional trigger, and psychiatric history). Nonetheless, coronary angiography remains the gold standard for diagnosis [17]. In our case, TTC was suspected on echocardiography but confirmed by angiography, as per current best practices.

Contrary to earlier beliefs, TTC is not a benign condition. While most patients recover LV function within weeks, the potential for serious complications, including arrhythmias, thromboembolism, and shock, demands vigilance [18]. As research evolves, future guidelines may standardize long-term follow-up, implantable devices in arrhythmic cases, and tailored therapies to prevent recurrence. Our case contributes to the growing recognition that even high-risk TTC patients can achieve favorable outcomes with timely diagnosis and comprehensive management.

## Conclusions

TTC should be considered in the differential diagnosis of ACS, particularly in older women with recent emotional or physical stress. However, it is important to recognize that TTC can also affect a broader demographic, including men and younger individuals, especially in the setting of intense physical or psychological stressors. This case illustrates that TTC may present in patients with traditional cardiovascular risk factors and can clinically mimic ACS, including biomarker and ECG profiles. Early coronary angiography, ideally within the first 24 hours, is essential to exclude obstructive coronary artery disease and confirm the diagnosis in alignment with ACS protocols. Echocardiography remains a valuable early tool in identifying the hallmark wall-motion abnormalities (e.g., apical akinesis with basal hyperkinesis). At the same time, cardiac MRI may be reserved for cases where echocardiography and angiography are inconclusive. In our patient, early echocardiography and later cardiac MRI confirmed transient ventricular dysfunction with subsequent recovery. Long-term follow-up is essential, particularly in patients with comorbidities such as diabetes, as they may be at increased risk for recurrence, persistent myocardial dysfunction, or arrhythmias. Suggested monitoring includes repeat echocardiography (e.g., at three to six months) to reassess left ventricular systolic and diastolic function, Holter monitoring in those with QTc prolongation or palpitations, and psychological evaluation to manage ongoing stressors. Residual diastolic dysfunction, as observed in our case, may reflect either TTC sequelae or pre-existing metabolic factors, underscoring the need for structured follow-up imaging to differentiate between transient and chronic abnormalities.
